# Anterior cingulate cortex connectivity is associated with suppression of behaviour in a rat model of chronic pain

**DOI:** 10.1177/2398212818779646

**Published:** 2018-06-05

**Authors:** Laurel S. Morris, Christian Sprenger, Ken Koda, Daniela M. de la Mora, Tomomi Yamada, Hiroaki Mano, Yuto Kashiwagi, Yoshichika Yoshioka, Yasuhide Morioka, Ben Seymour

**Affiliations:** 1 Department of Psychology and Behavioral and Clinical Neuroscience Institute, University of Cambridge, Cambridge, UK; 2 Center for Information and Neural Networks, National Institute of Information and Communications Technology, Osaka, Japan; 3 Computational and Biological Learning Laboratory, Department of Engineering, University of Cambridge, Cambridge, UK; 4 Pain & Neuroscience, Drug Discovery & Disease Research Laboratory, Shionogi & Co., Ltd., Osaka, Japan; 5 Translational Research Unit, Biomarker R&D Department, Shionogi & Co., Ltd., Osaka, Japan; 6 Immunology Frontier Research Center, Osaka University, Osaka, Japan; 7 Brain Information Communication Research Laboratory Group, Advanced Telecommunications Research Institute International, Kyoto, Japan

**Keywords:** Chronic pain, confirmatory factor analysis pain model, resting state functional magnetic resonance imaging, pain in rodents

## Abstract

A cardinal feature of persistent pain that follows injury is a general suppression of behaviour, in which motivation is inhibited in a way that promotes energy conservation and recuperation. Across species, the anterior cingulate cortex is associated with the motivational aspects of phasic pain, but whether it mediates motivational functions in persistent pain is less clear. Using burrowing behaviour as an marker of non-specific motivated behaviour in rodents, we studied the suppression of burrowing following painful confirmatory factor analysis or control injection into the right knee joint of 30 rats (14 with pain) and examined associated neural connectivity with ultra-high-field resting state functional magnetic resonance imaging. We found that connectivity between anterior cingulate cortex and subcortical structures including hypothalamic/preoptic nuclei and the bed nucleus of the stria terminalis correlated with the reduction in burrowing behaviour observed following the pain manipulation. In summary, the findings implicate anterior cingulate cortex connectivity as a correlate of the motivational aspect of persistent pain in rodents.

## Introduction

One of the greatest challenges in the development of novel analgesics is the difficulty in evaluating pain in animal models of chronic pain. Pain is a uniquely subjective experience, and the mainstay of outcome measures in clinical trials remains subjective ratings. Since these are not available in animals, evaluation of pain depends on surrogate measures of behaviour, primarily motor responses directly related to evoked or spontaneous pain. However, the difficulty in translating these behaviours to humans is well recognised, and this greatly hampers the ability to predict whether analgesics that are successful in animals will work in humans. This has led to a requirement for behavioural measures that better reflect persistent pain in animals, and which have translational validity to humans.

Functional neuroimaging offers a novel approach to the evaluation of pain in rodents (Baliki et al., 2014; Thompson and Bushnell, 2012). In humans, a broad set of cortical and subcortical regions are implicated in pain processing and the expression of pain behaviour. This diversity reflects the fact that pain is a multidimensional experience, engaging sensory, affective, and cognitive processing. Human studies of phasic experimental pain implicate a network of pain-related regions that includes thalamus, primary and secondary somatosensory cortex, cerebellum, the insular cortex, and the anterior cingulate cortex (ACC) (Moisset and Bouhassira, 2007; Price, 2000). Although the precise functions of each region remain unclear, there is a consensus that some regions (thalamus, primary and secondary somatosensory cortex, cerebellum) better reflect sensori-motor processes, and others (the anterior insular cortex and in particular the ACC) better reflect affective processes related to pain (Büchel et al., 2002; Morrison et al., 2004; Rainville et al., 1997). However, there remains uncertainty as to how well studies of phasic experimental pain translate to the persistent pain seen in chronic pain models. Although phasic pain occurs as a part of many chronic pain conditions, as seen with spontaneous phasic and hyper-sensitive evoked pain, the nature of persistent underlying pain is quite different and serves a distinct behavioural function to reduce activity and conserve energy.

The ACC is a strong candidate to play a dominant role in mediating the affective behavioural manifestations of persistent pain. It serves a primary function as a modulator of the affective tone of visceral, motor and endocrine efferents to downstream regions involved in responses to nociceptive stimuli, including the periaqueductal grey (PAG), the amygdala (Calejesan et al., 2000; Vogt et al., 1992; Zhuo, 2007), and the basal forebrain (Kash et al., 2015).

Recently, novel behavioural tasks such as burrowing behaviour (BB) have been proposed as a measure of the affective impact of pain. BB is an innate, spontaneous behaviour that demonstrates the overall ‘wellbeing’ or affective-motivational tone of the rats (Deacon, 2006) and is not affected by limb hypersensitivity (Andrews et al., 2012). Burrowing provides shelter and protection from environmental predators, food storage, and foraging, but also comes with energy costs (Reichman and Smith, 1990). This behaviour is reduced in rats following peripheral nerve injury or pain (Andrews et al., 2012), and therefore may provide a useful index of the motivational component of persistent background pain.

Taken together, these considerations suggest that changes in ACC connectivity may be related to the modulation of motivational behaviours, such as burrowing, by persistent pain. To test this hypothesis, we studied BB in adult rats in a model of inflammatory arthritis pain (intra-articular injection of complete Freund’s adjuvant) and measured ACC functional connectivity in the brain using functional magnetic resonance imaging (fMRI).

## Materials and methods

### Experimental animals

Seventy-two male LEW/CrlCrlj rats (Charles River Laboratories, Japan, Inc.) were used. Rats were housed in groups of three in plastic cages under controlled temperature and humidity and provided free access to food and water under a 12/12 h reversed light-dark cycle (lighting at 8:00 a.m.). To reduce transfer stress (single housing, changing the cage-mates) and negative impact (active social interaction or attacks from non-injured rats) from non-injured rats to injured rats, the rats were housed and transported only with rats from their group. All procedures were approved by internal animal care and use committee of Shionogi Pharmaceutical Research Center (Osaka, Japan) instructed by Association for Assessment and Accreditation of Laboratory Animal Care International (AAALAC) guidelines.

### Preparation of pain model rats

Rats were anesthetised with isoflurane (Mylan, Canonsburg, PA, USA) and then intra-articular injected with 25 µL of either Freund’s Complete Adjuvant (confirmatory factor analysis (CFA); *Mycobacterium butyricum* (BD DIFCO, Franklin Lakes, NJ, USA); 2 mg/mL of liquid paraffin (Maruishi Pharmaceutical Co., Ltd., Osaka, Japan)), or vehicle (sham rats) into the right knee joint of the hind leg. In both cases, the left hind knee joint was kept untreated. Both the pain model and the sham rats were 6 weeks old at the time of injection.

### Pain behaviour

Rats were tested for pain behaviour 18 and 22 days after CFA/vehicle injection. First, the knee diameter (KD) was determined using a digital caliper (CD-15CX; Mitutoyo Corporation, Kanagawa, Japan). Second, a weight bearing difference (DWB) test on each hind leg was performed (Bioseb, Boulogne, France). Values of WBD are obtained using the following formula, where W_R_ corresponds to the amount of weight put on the right leg and W_L_ to the amount of weight put on the left leg

$$\%W_{R}=100\times W_{R}/\left(W_{R}+W_{L}\right)$$

This formula expresses the percentage of the rat’s body weight put on the CFA/vehicle-injected right leg; thus, a 50% value means equal weight distribution across both hind legs.

Third, a grip strength (GS) test (San Diego Instruments, San Diego, CA, USA) was conducted to quantify the mechanical strength of the hind legs and hence to control for the development of pain in model rats. Briefly, each rat was gently restrained and allowed to grasp the wire mesh frame with its hind limbs and was moved in a rostral-to-caudal direction until the grip released. Finally, BB was investigated. For burrowing experiments, plastic tubes (32 cm in length and 10 cm in diameter) were filled with 3 kg of gravel (5–8 mm particle size) and placed in Plexiglas cages (560 × 440 × 200 mm). The open-end of the tube was elevated 6 cm from the floor of the cage. Rats were allowed to individually burrow during 30 min for 18 days after CFA or vehicle injections were performed and the amount of gravel burrowed was recorded.

For the statistical comparison of the behavioural data from the CFA pain model and control animals, we employed a two-sample student’s t-test (one-tailed). Results were considered significant at p < 0.05.

### Animal selection and transfer to magnetic resonance imaging facilities

Based on the results obtained from the WBD on the 18th day, rats were selected out of the ones that displayed either moderate or severe pain and were sent to the magnetic resonance imaging (MRI) facility. Selection criteria for CFA rats were <35% of WBD value. Functional MRI scanning was conducted at the Center for Information and Neural Networks (CiNet, Osaka University, Suita, Japan). After being transferred to the MRI facility, animals were kept for 4 days under standard laboratory conditions (room temperature of 22°C–23°C and a 12 h light/dark cycle) with free access to food and water. Rats were group housed with previous cage mates during and after transportation. MRI data were acquired on the third day (i.e. 21 days after CFA/vehicle injection). On the fourth day, behavioural data were obtained once more.

### Resting state functional MRI data acquisition

Data was acquired from a total of 37 rats (18 CFA pain model) with an 11.7 Tesla Avance II vertical bore system (Bruker BioSpin, Ettlingen, Germany) and a home-made transmit/receive surface radio frequency (RF) coil. Rats were anesthetised with a mixture of air and 2.8% isoflurane (Wako Pure Chemical Industries Ltd., Osaka, Japan) and then placed in an MRI-compatible animal cradle. The isoflurane concentration was maintained at 2% ± 0.5%, adjusted to maintain the respiration rate at 70 ± 10 breaths per min throughout the sessions.

An axial T2-weighted (T2W) imaging was performed using a rapid acquisition of relaxation enhancement (RARE) sequence (repetition time/echo time (TR/TE) = 6500/45 ms, number of averages (NA) = 8, field of view (FOV) = 32 × 16 mm, matrix size = 256 × 256, slice thickness = 500 µm, acquisition time = 14 min).

To acquire resting state functional magnetic resonance imaging (rsfMRI) data, we performed gradient-echo echo-planar imaging (TR/TE = 2500/7 ms, number of segments = 2, flip angle = 60°, FOV = 51.2 × 51.2 mm, matrix size = 64 × 64, slice thickness = 1 mm, in-plane resolution = 800 × 800 µm^2^, bandwidth = 300 kHz, acquisition time = 20 min).

### Imaging data preprocessing

The imaging data preprocessing was performed with AFNI (https://afni.nimh.nih.gov) and FSL (FMRIB, University of Oxford, UK; https://fsl.fmrib.ox.ac.uk/fsl/fslwiki/MELODIC).Data from seven animals could not be analysed due to technical difficulties and resulting poor image quality leaving 30 animals for the final rsfMRI analysis (14 animals with pain, 16 controls). Functional data were corrected for slice timing offsets, with mean and linear detrending (3dTshift) and images were realigned to a single middle slice (3dvolreg) for volume-to-volume rigid body correction. Resultant motion and displacement parameters were visually inspected for outliers. Anatomical data were skull stripped with a standard spherical model (3dSkullStrip). In order to do this skull strip, anatomical data were ‘shrunk’ in the anterior–posterior direction (3drefit) to make the brain more spherical, in a manner similar to (Kundu et al., 2014). After effective skull strip, anatomical data were returned to their original voxel dimensions. Functional and anatomical data were then roughly aligned in space (@align_centers) and orientation when oblique (3drotate). Functional data were up-sampled to match anatomical voxel dimensions (3drefit). Anatomical and functional data were then coregistered using a 12-paramter registration (3dAllineate). A study-specific anatomical template was created by averaging four normal control rat anatomical datasets. Anatomical data for each rat were normalised to this study-specific template and resultant warp parameters were applied to their respective functional data (3dAllineate). Functional data were furthermore subjected to de-spiking (3dDespike), smoothing with full width at half maximum = 0.5 mm, band pass filtering (0.01 < f < 0.1), and linear regression of motion parameters (3dTproject).

### Assessment of functional connectivity

Functional connectivity (Friston, 1994) of the ACC was assessed with a seed-based functional connectivity approach. The ACC seed region was determined as the anterior portion of Brodmann’s area 24 (Vogt and Peters, 1981), approximately 1 mm rostral to Bregma (Calejesan et al., 2000). The mean time series in this region were extracted for each animal from a sphere of 0.5 mm radius around this coordinate and functional connectivity was determined by calculating brain-wide z-transformed correlation maps based on the preprocessed functional time series. Subsequently, functional connectivity maps were compared between groups employing a two-sample t-test. Finally, correlations between the individual functional connectivity maps and the behavioural measures (KD, DWB, GS, BB) were calculated across animals and compared between groups employing likewise t-statistics.

Cluster correction with AFNI’s 3dClustSim (Cox et al., 2017) was used to reduce the instance of false positives caused by spatial autocorrelation suggested by Eklund et al. (2016) (p < 0.05 per voxel plus cluster size k = 15,131 voxel, corrected at alpha < 0.01).

## Results

### Behavioural and physiological measures

**Figure 1.**
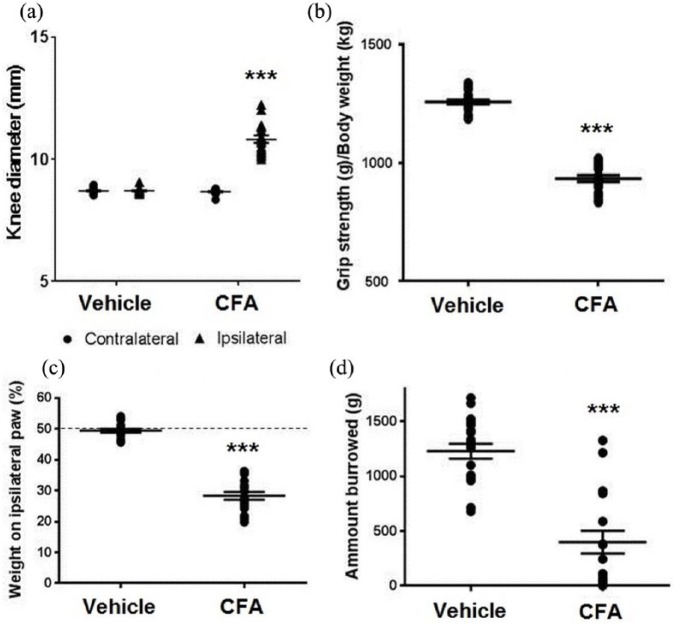
Behavioural and physiological changes of CFA pain model rats compared to controls at the time of fMRI scanning. (a) Changes in contralateral and ipsilateral knee diameter (KD) in vehicle and CFA rats at day 22. (b) Changes in hind limb grip strength (GS) in vehicle and CFA rats. Data are shown as an average value of days 18 and 22 expressed as grip strength/body weight. (c) Changes in dynamic weight bearing (WBD) on the ipsilateral paw in vehicle and CFA rats. Each data were shown as an average value of days 18 and 22. (d) Changes in burrowing behaviour (BB) in vehicle treated and CFA pain model rats at day 18. *** indicates p < 0.001.

KDs were measured to determine the amount of joint swelling as an index of inflammation. Ipsilateral KD in CFA pain model rats was significantly increased compared to vehicle-treated rats at day 22 (mean KD CFA-treated rats 10.8 ± 0.15 (SEM), mean KD control group 8.7 mm ± 0.03, t(28) = 14.2, p < 0.001; Figure 1(a)). CFA pain model rats also showed decreased GS (mean GS CFA animals 931.0 g/kg ± 30.1, mean GS control group 1259.0 g/kg ± 10.6, t(28) = 17.4, p < 0.001; Figure 1(b)), weight on ipsilateral paw (mean DWB CFA-treated rats 27.7% ± 1.5, mean DWB control group 49.5% ± 0.7, t(28) = 13.9, p < 0.001, Figure 1(c)), and amount of burrowed gravels (mean BB CFA-treated rats 444.5 g ± 121.5, mean BB control group 1238.2 g ± 72.2, t(28) = 5.8, p < 0.001, Figure 1(d)). The results suggest that CFA pain model rats showed evoked and spontaneous inflammatory knee joint pain at the time of fMRI scanning.

### Resting state functional connectivity

**Figure 2.**
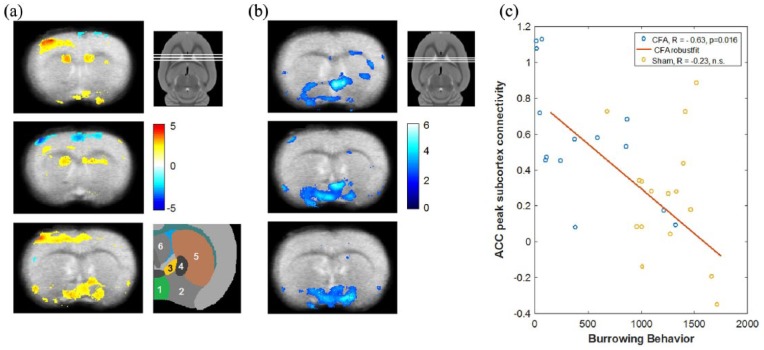
Changes in resting state functional connectivity in CFA pain model rats compared to control and correlation with burrowing behaviour. (a) Changes in ACC resting state functional connectivity in CFA pain rats compared to vehicle-treated animals. The ACC showed increased functional connectivity in CFA pain rats with parts of the contralateral somatosensory cortex (upper and lower panels), the bilateral striatum (upper and middle panels) and with the basal forebrain region/preoptical area and the region of the bed nucleus of the stria terminalis (lower panel). ACC functional connectivity was decreased in parts of the cingulate cortex itself and in the somatosensory cortex (middle panel). The three white lines in the smaller panel next to the upper panel of (a) indicate the approximate rostro-caudal position of the three axial sections of (a) in the rat brain. The colour bar indicates t values. Hot colours represent increased functional connectivity and cold colours represent decreased connectivity. A schematic representation of the involved subcortical regions is shown next to the lower panel of (a). (1) Hypothalamic/preoptical area, (2) basal forebrain region, (3) bed nucleus of the stria terminalis, (4) globus pallidus, (5) striatum, (6) septal region. (b) Negative correlation of burrowing behaviour with the individual strength of ACC functional connectivity. The colour bar indicates t values. The visualisation threshold is set to p < 0.005 uncorrected in (a) and (b). Note the spacial overlap of increased ACC-subcortical connectivity in (a) with the negative connectivity–behaviour correlation in (b) and the slightly different positions of the axial sections in (b) compared to (a). (c) Illustration of the negative correlation between burrowing behaviour with the individual connectivity strength at the subcortical peak in the upper panel of (b). ACC connectivity shows a marked negative correlation with burrowing in the CFA pain group but not in the control group.

The ACC seed region showed reduced functional connectivity in the CFA pain group compared to control animals with central parts of the contralateral somatosensory cortex and dorsal portions of the cingulate cortex (Figure 2(a)). In addition, the ACC exhibited increased functional connectivity in the pain group with rostral portions of the somatosensory cortex and in the subcortex with the bilateral striatum, structures of the basal forebrain region, hypothalamic region/preoptical area (POA), and the area of the bed nucleus of the stria terminalis (BNST; see Figure 2(a)).

In CFA pain animals, we found a marked negative correlation between ACC functional connectivity and innate BB in the latter regions (POA & BNST; Figure 2(b)), that is, higher functional connectivity between ACC and these regions was associated with suppression of BB.

For illustration purposes, Figure 2(c) shows the correlation of connectivity strength between ACC with the peak subcortical region of interest and BB. ACC connectivity shows a negative correlation with burrowing in the CFA pain group (r = –0.63, p = 0.02) but not in the control group (r =−0.23, p = 0.39). The interaction failed, however, significance (z = 1.2, p = 0.11). We found no significant correlations between ACC connectivity with KD, weight bearing, and GS.

## Discussion

In the present study, we assessed spontaneous BB in the CFA model of persistent inflammatory arthritis pain and investigated ACC resting state functional connectivity compared to controls. The behavioural measures consistently indicated inflammatory nociception related to the CFA-treated joint at the time of MRI measurement by showing ipsilateral swelling, reduced motoric ability, and reduced weight bearing. As hypothesised, we also observed reduced BB accounting on average for less than 50% of the behaviour in control animals. Resting state fMRI revealed a pronounced negative correlation between the individual strength of ACC functional connectivity and BB in structures of the basal forebrain, comprising the hypothalamic region and the BNST. No correlations with the ACC functional connectivity were observed for motor responses directly associated with joint pain. The findings therefore indicate a relatively specific relationship between ACC functional coupling and the suppression of BB by persistent pain and suggest that ACC connectivity might be a good marker for the affective-motivational component of pain in rodents.

Across mammalian species neuronal responses to noxious stimuli have been shown to involve a number of brain regions including the primary and secondary somatosensory cortex, the ACC, and the insular cortex (Iadarola et al., 1998; Lenz et al., 1998; Peyron et al., 2000; Ploner et al., 2002; Pohlmann et al., 2016; Thompson and Bushnell, 2012).

Although the precise contribution of each region to the generation of pain remains to be elucidated (Moayedi and Davis, 2012; Segerdahl et al., 2015), neuroimaging studies highlighted some general characteristics of the involved regions. While the somatosensory cortex responds faster to dimensional features of noxious stimuli, and is more engaged when larger body surfaces are exposed to pain, the ACC as part of the limbic system is thought to be modulated by the affective relevance pertinent to a change in motivational tone or response selection (Peyron et al., 2000; Ploner et al., 2002).

The ACC is often conceptualised as a nexus for the processing of external salient stimuli, autonomic response regulation, and subsequent affective learning (Gao et al., 2004; Vogt, 2005). For example, fear learning in mice through observing other mice receiving painful foot shocks has been demonstrated to involve the ACC (Jeon et al., 2010). In the context of pain, the ACC is thought to allow potentially harmful stimuli to engage appropriate affective and motivationally relevant behaviours, and as such one would expect the strength of ACC-based connectivity to be associated with elevated aversive behaviors and reduced behaviours of well-being. Spontaneous BB in rodents is considered as one of the latter (Deacon, 2006), and hence, the association of higher connectivity between ACC and subcortex with reduced burrowing is consistent with a possible functional inhibitory pathway.

While the association between ACC-hypothalamic coupling and BB might reflect a general homeostatic dimension related to the animal’s behavioural suppression, the correlation between burrowing and coupling strength with the BNST is especially interesting as it has been linked to sustained vigilance associated with ambiguous or distant threat cues. This potentially provides a direct link to the affective dimension of pain and illustrates BB as an expression of the affective-motivational tone of the animals.That is, when a limb is injured, the potential danger by predators and accordingly monitoring of potential threats becomes much more important, so that behaviours such as burrowing are no longer prioritised. In line with this, lesions of the BNST have been demonstrated to disrupt the individual variability in the rodent’s anxiety-like behaviour (Durvarci, 2009). Finally, the role of the BNST as a site of integration of limbic forebrain information is also supported by tracing studies showing direct anatomical BNST connections with the ACC (area 32) (Kash et al., 2015).

The findings have implications for pain testing in animals. Most tests of pain in chronic pain models evaluate evoked pain, in which enhanced defensive behaviours are observed in response to stimulation of some sort. Such tests are likely to be highly sensitive to hyperalgesia and allodynia, but less so to the overall decrease in ‘wellbeing’ that accompanies it. Other tests, designed to capture persistent pain better, such as dynamic weight bearing, may still involve modulation of motor responses related to the pain associated with movement, but may be less likely to relate purely to affective-motivational component of persistent pain. In contrast, burrowing is an innate motivated behaviour, and in the case of hind leg CFA injection, modulation by pain is more likely to reflect the underlying affective suppression of behaviour in a way not directly related to exacerbation of the pain-inducing lesion. Given accumulating evidence that the transition to chronic pain can be characterised as the formation of a pathological affective state (Apkarian et al., 2013), pain tests related to the affective-motivational dimension of the sustained pain state might be more closely related to relevant pathophysiological changes associated with disease progression. That suppression of BB relates to ACC connectivity, which is strongly implicated in affective components of pain, further supports this notion, and adds to evidence that burrowing may provide a valuable complement to conventional measures in the evaluation of pain in rodent models of chronic pain. Assessing motivational parameters in the context of sustained pain in animals also captures information that has greater relevance to general well-being and higher-order biological goals compared to purely local measures, which is in line with the recent notion that more natural animal models might have a better translational validity (Klinck et al., 2017) and likewise with the finding that also in humans local parameters have only limited prognostic value.

Several limitations of the study should be noted. Experiments were conducted in anesthetised animals and we cannot exclude that the narcotic agent interfered to a certain extent with the involved nociceptive mechanisms (Tsurugizawa et al., 2016) or the observed association between resting state connectivity and suppression of behaviour. However, even high concentrations of isoflurane exert only mild antinociceptive effects and reexamination of the behavioural measures after fMRI measurements showed no significant changes. Anaesthetics naturally interfere with the brain-wide spatiotemporal organisation of functional networks, but it appears very unlikely that a narcotic agent selectively induced associations between spontaneous fluctuations of resting state brain activity and behavioural measures in one group of animals.

Notwithstanding, neuroimaging experiments alone are inherently correlational, and it cannot be assumed that the ACC connections mediate a direct causal suppression of affective-motivational behaviour on the basis of the evidence provided. Even if the connectivity is indeed causal, one needs to remain cautious about generalising to other motivated behaviours, in case the pathway was specific to BB. With this in mind, it is difficult to find an exact parallel to BB in humans. However, these issues can be potentially addressed. For instance, the suppression of affective and motivated behaviours could be studied in human pain patients with regard to ACC connectivity.

The employed experimental paradigm also provides interesting perspectives for future studies in rodents. Pharmacological studies using systemic analgesics could investigate correlations between suppression/reinstatement of BB, ACC connectivity, and the development of the painful condition. And targeted manipulations of the ACC (local pharmacology, optogenetics) with concurrent neuroimaging could be highly informative with regard to the identification of potential causal relationships.

## Supplemental Material

supp-rat – Supplemental material for Anterior cingulate cortex connectivity is associated with suppression of behaviour in a rat model of chronic pain Click here for additional data file. Supplemental material, supp-rat for Anterior cingulate cortex connectivity is associated with suppression of behaviour in a rat model of chronic pain by Laurel S. Morris, Christian Sprenger, Ken Koda, Daniela M. de la Mora, Tomomi Yamada, Hiroaki Mano, Yuto Kashiwagi, Yoshichika Yoshioka, Yasuhide Morioka and Ben Seymour in Brain and Neuroscience Advances
