# Supplementary figures and images for: Anterior cingulate cortex connectivity is associated with suppression of behaviour in a rat model of chronic pain
Source: Brain Neurosci Adv. 2018 Jun 5;2:2398212818779646. doi: 10.1177/2398212818779646 (PMC6109941; doi:10.1177/2398212818779646)

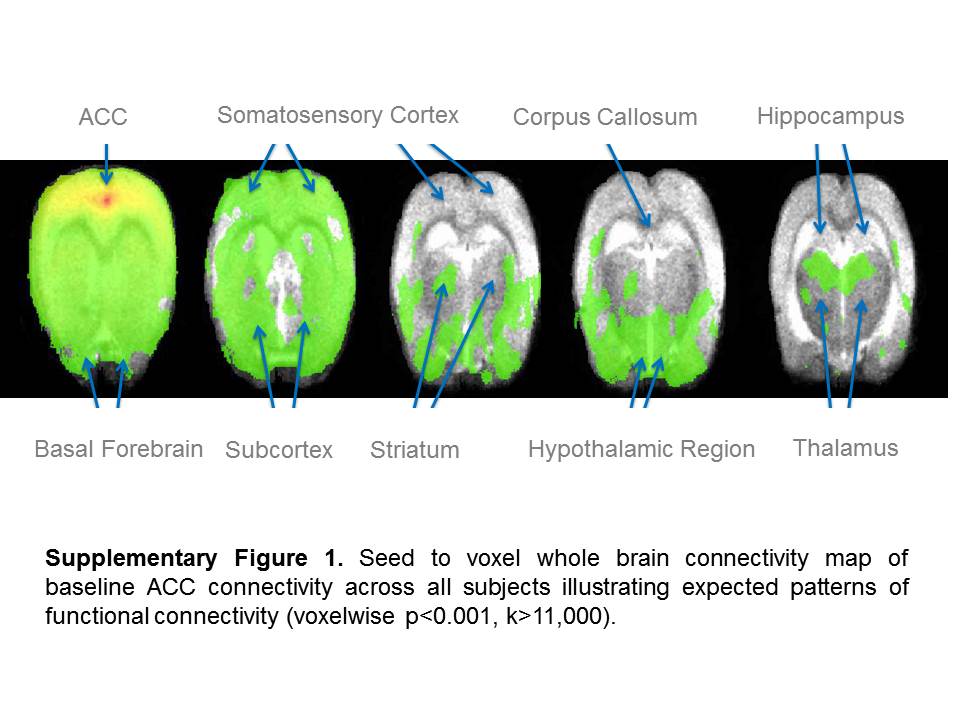

Supplement: supp-rat – Supplemental material for Anterior cingulate cortex connectivity is associated with suppression of behaviour in a rat model of chronic pain [file supp-rat.jpg]
